# Why is Zika virus so rarely detected during outbreaks and how can detection be improved?

**DOI:** 10.1186/s13104-017-2854-8

**Published:** 2017-10-30

**Authors:** Diawo Diallo, Mawlouth Diallo

**Affiliations:** Unité d’Entomologie Médicale, Institut Pasteur de Dakar, 36 Avenue Pasteur, BP 220, Dakar, Senegal

**Keywords:** Zika, Arbovirus, Outbreaks, vectors, Field-collected mosquitoes

## Abstract

**Objective:**

Even during outbreaks, detection of Zika virus (ZIKV; genus *Flavivirus*, family *Flaviviridae*) in its mosquito vectors is surprisingly uncommon. Here we explore the reason for this apparent paradox and suggest strategies for improving the efficacy of ZIKV detection.

**Results:**

There are several likely explanations for the rarity of ZIKV detection in field-collected mosquitoes during outbreaks, including the lag between the period when people are clinically ill and the initiation of entomological investigations, the prompt spraying of houses of identified cases, the difficulty of identifying some of the households of ZIKV infected cases, and the low efficiency of the sampling methods currently available. Thus, timely entomological investigation of suspected cases before the intervention of the vector control squad would enhance ZIKV detection from mosquitoes. For this to happen, administrative, financial and logistical issues must be solved before the beginning of outbreaks, and routine entomological surveillance must be conducted in foci of ZIKV amplification. Improving ZIKV detection during outbreaks is of paramount importance because identification of the mosquito species and population involved as vector in a given outbreak is a key element to a comprehensive and effective vector control strategy.

## Introduction

Mosquito-borne Zika virus (ZIKV; genus *Flavivirus*, family *Flaviviridae*) has spread globally in recent years. Until 2006, this arbovirus was only known in Africa and Asia and considered of minor interest [1, 2]. During this period, transmission occurred mainly in a sylvatic cycle involving arboreal *Aedes* and non-human primates [3]. Only a few human cases have been described in this sylvatic cycle with mild symptoms including rash, joint pain and conjunctivitis [2]. The first major outbreak of ZIKV was observed on Yap Island in 2007 [1], thereafter outbreaks occurred on several islands in the Pacific including French Polynesia in 2013 and 2014 [4, 5]. Nevertheless, this virus really attracted world attention when it arrived in South America in 2015. Indeed, in Brazil alone, more than 1,300,000 suspected cases were recorded including cases with neurological complications like Guillain-Barré syndrome and more than 3000 cases of microcephaly in newborns of women infected during pregnancy [6, 7]. To date, over 84 countries and territories have experienced local mosquito-borne transmission of ZIKV, and imported cases were recorded in many other countries in Europe and Americas [8]. Although cases of sexual and congenital transmission of ZIKV have been reported, ZIKV is likely transmitted between humans mainly by the mosquito *Ae. aegypti* [9] and to a lesser extent by *Ae. albopictus* [10]. Indeed, only these two species have been found naturally infected with ZIKV during outbreaks [10–17]. In addition, they are the only vectors with ecological opportunity to maintain outbreaks that have also been shown to transmit the virus in laboratory assays [18–20]. Yet despite the increase in the number, frequency and severity of ZIKV outbreaks, the virus has rarely been detected in mosquitoes in the vicinity of these outbreaks. Indeed, ZIKV was detected from mosquitoes collected during outbreaks only in the following four countries: Gabon (from *Ae. albopictus* in 2007 [10]), Mexico (from *Ae. aegypti* in 2015–16 [11, 16]), Brazil (from *Ae. albopictus* adults in 2016 [17], from *Ae. aegypti* adults in 2015–16 [12, 15] and *Ae. albopictus* eggs in 2015 [14]), and Singapore (from *Ae. aegypti* and *Ae. albopictus* in 2016 [13]). Several entomological investigations following outbreaks in Yap Island [21], French Polynesia, Brazil [22], American countries, Cabo Verde, Guinea Bissau (Diallo et al. unpublished data) in which several mosquito species were collected from the field and tested by real-time RT-PCR and/or virus isolation attempts failed to detect ZIKV. This paucity of detection is surprising for a primarily vector-borne disease. The reasons for low detection rates must be identified and addressed, because the identification of the mosquito species involved as vectors in a given outbreak is a key element to a comprehensive and consolidated action plan for vector control.

The aim of this paper is to present some probable explanations of the rarity of ZIKV detection from mosquito vectors collected in the vicinity of ZIKV outbreaks.

## Main text

The overall objective of the entomological investigation of an outbreak is to collect data on mosquitoes (including their identity, abundance, behavior and infection status) in and around households of infected patients as well as the path of their movements during the period of host viremia. These data are used to inform decisions on an effective vector control strategy.

Thus, failure to detect ZIKV during outbreaks significantly hinders ZIKV control. There are at least four explanations for the lack of ZIKV detection in field-collected mosquitoes during outbreaks:

## Delay between identifying illness in people and conducting field investigations

In the few ZIKV outbreaks investigated by entomologists, investigations were sometimes conducted several months after the detection of the outbreak. In the first large ZIKV outbreak in Yap in 2007, there was a delay of about 3 months between the beginning of the outbreak in April and the entomological investigation in July [21]. In the recent ZIKV outbreaks observed in Africa, this delay was 7 months in Cabo Verde (first human cases detected in October 2015 and the beginning of the entomological investigation at the end of March 2016) and 3 months in Guinea Bissau (first human cases detected in may 2016 and the entomological investigation done in August 2016) (Diallo et al. unpublished data). If we take into account the short lifespan of female mosquitoes including *Ae. aegypti*, investigations are generally made after turnover of the population responsible for the outbreak. Moreover, some investigations are conducted much later, when mosquito populations are at their lowest level or have completely disappeared as a result of the installation of seasonal changes in weather. ZIKV was detected from field-collected mosquitoes during outbreaks when entomological investigations were made in and around households of clinically ill patients, indicating that the transmission was still ongoing [10–13]. For the first detection of the virus in Brazil, mosquitoes were collected during the same week as clinical diagnostic of the patients [12]. All others detections from field-collected mosquitoes were from samples collected during routine entomological surveillance of arboviruses [3, 9, 16, 23, 24].

Delay in entomological investigations in response to outbreaks is primarily attributable to delays in diagnosis and reporting of cases due to lack of knowledge of the disease by physicians, high proportion of asymptomatic cases, and the lack of rapid diagnostics for flavivirus infections [25]. Other reasons for delays include lack of experts in medical entomology [26, 27] as well as administrative and financial barriers. Medical entomologists are mainly trained in Masters and Ph.D. programs in relatively few universities and research institutions around the world. Only a small proportion of these entomologists have specific expertise in arboviruses. Thus, there is an urgent need to train and employ individuals with the skills to conduct entomological investigations in response to arbovirus outbreaks in all countries within the sphere of ZIKV transmission risk. Administrative issues that delay outbreak investigations include inadequate training of management staff, lack of coordination, lack of procedures or excessively complex procedures for decision making, data collection and transmission, reluctance to share data at the national and international level, and the low priority given to unknown, rare and unplanned events. Little, and in some cases no, national funding is dedicated to outbreak investigations in many countries. Emergency funding may be available via international agencies like World Health Organization but this must be requested by national authorities and the time required to process and consider such requests further delays the investigation.

## Difficulty in identifying Zika-infected households

The second explanation for failure to detect ZIKV in mosquitoes is the difficulty of identifying the exact households of ZIKV infected cases. Sampling mosquitoes within ZIKV case households increases the probability of detecting infected mosquitoes because *Ae. aegypti* females are mainly endophilic with very limited spatial dispersal, often spending their entire lives in the room where they emerged [28]. An entomological investigation in randomly selected houses failed to detect ZIKV in mosquito in Yap [21], while positive pools were detected when mosquito were collected in and around clinically ill patient households [10–12]. During outbreak investigations, households of Zika patients are identified via the addresses or phone numbers found in medical registers or patient identification forms sent by physicians to the laboratory along with samples for diagnosis. However, only around 20% of these ZIKV infected people are symptomatic [2], and an even smaller fraction seek medical care and thus may be identified and investigated through this system. Moreover, in many cases basic location information (exact address and phone number) are not reported in medical documents. Thus, it is impossible to identify and investigate the majority of households in which a ZIKV-infected individual lives. Further, it is not certain that a given patient was infected in his/her own household because infections could have been contracted in other neighborhoods or villages during the course of normal movements. To alleviate this problem, medical personnel must be instructed to clearly record the address, phone number and travel history in each patient identification form.

## Inefficient sampling methods

The third explanation of the scarcity of ZIKV detection in field collected mosquitoes is the low efficiency of the sampling methods currently available and commonly used for collecting populations of *Aedes* vectors [29, 30]. The collection of host seeking females by human landing catch is still probably the most effective method for *Ae. aegypti* and for sylvatic ZIKV vectors, but this method raises some ethical concerns. Humans used as collectors are exposed to the bite of potentially infective mosquitoes especially during epidemics [31]. People who have already been in contact with the virus (IgG or IgM positives) could be used as mosquito collectors because they are protected against ZIKV, but they are still exposed to other arboviruses and pathogens transmitted by mosquitoes. Other methods for collection include CDC light traps, backpack aspirators, resting site collections, BG-sentinel traps and various gravid traps; each comes with specific limitations. *Ae. aegypti* is not very attracted to the CDC light traps. The weight of the backpack aspirator makes it difficult to use and its performance depends on the competence and motivation of the operator [32]. It is extremely difficult to identify *Aedes* resting sites, making it difficult to collect an adequate number of mosquitoes. BG-sentinel traps have been shown to effectively collect *Ae. aegypti* [33] and *Ae. albopictus* [34]. However, data about their efficiency for sylvatic vector species and during ZIKV outbreak investigations are lacking.

ZIKV and many other flaviviruses infect mosquitoes at low rates, thus, to detect these viruses is necessary to collect a very large number of mosquitoes. Using statistical models, Gu et al. [35] have described methods to estimate the probability of arbovirus detection in mosquito populations. They showed that detection of low levels of mosquito infections requires large samples (greater than 1600 individuals) for a high (80%) probability of detection. They also indicated that grouping samples over different sampling sites and times is inappropriate for detection of mosquito infection due to focal transmission of arboviruses. Adult ZIKV vectors have been collected during outbreaks using light traps, vacuum aspiration and gravid traps in Yap [21], aspiration in Brazil and Mexico [11, 12], gravid traps in Singapore [13] and human landing catch in Gabon [10]. ZIKV has been detected from adult mosquitoes collected during outbreaks by aspiration in Brazil and Mexico [11, 12], gravid traps in Singapore [13] and human landing catch in Gabon [10]. The virus was also detected from adult mosquitoes collected as eggs during the ZIKV outbreak in Brazil in 2015 [14]. Because ZIKV, chikungunya and dengue are transmitted mainly by *Ae. aegypti*, these limitations of sampling apply to all three viruses. In contrast, West Nile virus is transmitted by mainly ornithophilic and zoophilic mosquitoes. *Culex* are efficiently collected by a variety of trapping tools including dry-ice supplemented CDC light traps and CDC gravid traps [36, 37]. Development of new sampling tools that most closely mimic human landing catch is still needed for entomological investigation for ZIKV outbreaks. The utility of adding a heat source and human body shaped support to the BG sentinel trap should be explored.

## The prompt spraying of houses once cases are identified

The last explanation for the common failure to detect ZIKV in mosquitoes is that vector control is undertaken quickly during some outbreaks. As soon as a suspected case is detected, the vector control squad goes to spray within and around his/her household. These prompt reactions probably eliminate the infected population around identified human cases. Vector control squads should be trained for the basic skills needed for entomological investigation including collecting and handling mosquito samples for morphological identification and virological testing. This way surveillance can be implemented without delaying necessary public health interventions.

## Limitations

The main limitation of this discussion is the lack of published data on the entomological investigations of ZIKV outbreaks, especially when the virus was not detected in mosquitoes. Thus, proposed explanations described here were mainly drawn from our own experiences in the investigation of arbovirus outbreaks (including ZIKV) as experts that have been deployed by international health organizations in several African countries. To our knowledge this is the first comprehensive consideration of the barriers that curtail the efficacy of mosquito surveillance during ZIKV outbreaks. Some of these factors are universal (i.e. the low efficiency of CDC light traps for *Ae. aegypti*) whereas others will apply to only a subset of countries within the range of ZIKV transmission (i.e. specific administrative barriers). Moreover, we recognize that the solution to some of these barriers are political or administrative and that some intersect with protections of personal privacy issues and thus will not be easily implemented in all ZIKV affected countries.

## Abbreviations

ZIKV: Zika virus
RT-PCR: reverse transcriptase-polymerase chain reaction
